# Sex differences in host defence interfere with parasite-mediated selection for outcrossing during host–parasite coevolution

**DOI:** 10.1111/ele.12068

**Published:** 2013-01-10

**Authors:** Leila Masri, Rebecca D Schulte, Nadine Timmermeyer, Stefanie Thanisch, Lena Luise Crummenerl, Gunther Jansen, Nico K Michiels, Hinrich Schulenburg

**Affiliations:** 1 Department of Evolutionary Ecology and Genetics, Zoological Institute, Christian-Albrechts-University of KielGermany; 2 Department of Animal Evolutionary Ecology, Institute of Evolution and Ecology, University of TuebingenGermany; 3 Department of Behavioural Biology, University of OsnabrueckGermany

**Keywords:** *Bacillus thuringiensis*, Bateman's principle, *Caenorhabditis elegans*, ecological immunology, host–parasite coevolution, Red Queen hypothesis, sexual selection

## Abstract

The Red Queen hypothesis proposes that coevolving parasites select for outcrossing in the host. Outcrossing relies on males, which often show lower immune investment due to, for example, sexual selection. Here, we demonstrate that such sex differences in immunity interfere with parasite-mediated selection for outcrossing. Two independent coevolution experiments with *Caenorhabditis elegans* and its microparasite *Bacillus thuringiensis* produced decreased yet stable frequencies of outcrossing male hosts. A subsequent systematic analysis verified that male *C. elegans* suffered from a direct selective disadvantage under parasite pressure (i.e. lower resistance, decreased sexual activity, increased escape behaviour), which can reduce outcrossing and thus male frequencies. At the same time, males offered an indirect selective benefit, because male-mediated outcrossing increased offspring resistance, thus favouring male persistence in the evolving populations. As sex differences in immunity are widespread, such interference of opposing selective constraints is likely of central importance during host adaptation to a coevolving parasite.

## Introduction

The selection pressures parasites exert through direct negative effects on host fitness are often exacerbated by the parasites' potential for fast adaptation (e.g. Ebert 1998). Hosts therefore benefit from sexual reproduction and outcrossing, because it allows faster recombination of favourable alleles. This idea represents the central component of the Red Queen hypothesis (Hamilton 1980; Bell 1982; Lively 2010b) and has been well characterised with the help of mathematical models (e.g. Gandon & Otto 2007; Salathe *et al*. 2009; Lively 2010a; Hodgson & Otto 2012). Over the last years, the hypothesis has also found support from numerous empirical studies. For example, long-term studies of the freshwater snail *Potamopyrgus antipodarum* revealed an increased rate of sexual reproduction in the presence of its trematode parasite, and spatial and temporal adaptation to coevolving parasite varieties, both under field and laboratory conditions (Jokela *et al*. 2009; King *et al*. 2009, 2011; Koskella & Lively 2009). Similar support for the Red Queen hypothesis was obtained using the waterflea *Daphnia magna* (e.g. Ebert *et al*. 2007) or the red flour beetle *Tribolium castaneum* as model hosts (e.g. Kerstes *et al*. 2012).

All current examples focus on the high reciprocal selective pressures that the antagonists exert upon one another at the interspecific level. Intraspecific constraints may be of additional importance, especially if they directly affect the interaction with parasites. One such constraint is the difference among sexes in immunocompetence (i.e. the ability of an organism to defend itself against parasites). These differences may have several underlying causes, including sexual selection and Bateman's principle (Rolff 2002). The latter hypothesises that the sex with the higher reproductive potential, usually male, can predominantly increase fitness through an augmented mating rate, whereas the other sex, usually female, maximises fitness through longevity and thus an expanded time window for producing the more costly gametes. In response to these sex-specific trade-offs, females are predicted to invest more in immunity than males (Rolff 2002).

We studied the relevance of sex differences in immune defence during the evolutionary interaction with parasites using the nematode *Caenorhabditis elegans* as a model host. *C. elegans* has an androdioecious reproductive system defined by two genders, hermaphrodites and males. Hermaphrodites first produce sperm, which are stored for self-fertilisation of the subsequently produced eggs. Outcrossing is only possible between hermaphrodites and males. As male sperm outcompete hermaphrodite sperm (LaMunyon & Ward 1995), male–hermaphrodite matings result in complete cross-fertilisation and 1 : 1 sex ratios. Male offspring may also be produced through spontaneous X chromosome non-disjunction during meiosis, although usually at very low rates (Hodgkin *et al*. 1979; Hodgkin & Doniach 1997). Thus, male frequency is often taken as a direct indicator for the outcrossing rate in *C. elegans* populations (Teotónio *et al*. 2006, 2012). A recent evolution experiment indeed used this approach to demonstrate that coevolution with *Serratia marcescens* microparasites increased *C. elegans* outcrossing rates and, in turn, its ability to adapt to the antagonist (Morran *et al*. 2011, 2012). Our current study was based on a related interaction model, consisting of *C. elegans* and the Gram-positive bacterium *Bacillus thuringiensis*. This model previously allowed us to obtain experimental evidence for the manifold consequences of host–parasite coevolution, including reciprocal changes in parasite virulence and host defence, reciprocal life-history trade-offs, reciprocal increases in evolutionary rates and genetic diversity and an increase in local adaptation (Schulte *et al*. 2010, 2011, 2012).

In this study, we focused our analysis on male hosts and their interaction with parasites. The interspecific selection between coevolving hosts and parasites should favour male *C. elegans* because they are required for outcrossing in this species. At the same time, males may also be disfavoured in the presence of parasites, if they invest less in defence in response to intraspecific selective constraints. Therefore, males may be subject to a conflict between these inter- and intraspecific selective constraints. Our study was based on two approaches: (1) Two fully independent host–parasite coevolution experiments were used to analyse variation in male frequencies and thus outcrossing rates during the continuous coadaptation between the antagonists. (2) Separate focused experiments were performed to identify the traits that may selectively favour or disfavour males in the presence of parasites, such as male resistance, male sexual activity, male escape response and parasite resistance of male-derived outcrossed offspring. The combination of the two approaches allowed us to assess in how far sex differences in host defence influence outcrossing rates and thus the potential for adaptation to coevolving parasites.

## Materials and Methods

### Nematode and bacterial strains

Both evolution experiments and all subsequent tests were based on outbred, genetically diverse *C. elegans* populations. The starting population of the first evolution experiment was derived from reciprocal crosses among three natural isolates, as previously described (Schulte *et al*. 2010). For the second evolution experiment and all subsequent tests on male advantages and disadvantages, we used material from the genetically diverse and outbred population originally prepared by Henrique Teotónio through consecutive crosses among 16 natural isolates (Teotónio *et al*. 2012). This population was adapted to our experimental conditions over 10 generations in 40 replicates in the presence of the non-nematocidal *B. thuringiensis* strain DSM-350, to minimise the impact of selective constraints unrelated to host–parasite interactions during the following evolution experiment. The 40 pre-adapted populations were mixed, aliquoted and cryopreserved at −80 °C (Stiernagle 2006) for later usage. Worm maintenance otherwise followed standard procedures (Stiernagle 2006).

For *B. thuringiensis*, identical genotype mixtures were used as starting material for both evolution experiments and all subsequent tests, including the three nematocidal strains MYBT18246, MYBT18247 and MYBT18679 (Schulte *et al*. 2010). The non-nematocidal *B. thuringiensis* DSM-350 served as a control. Prior to experiments, *B. thuringiensis* was cultured in large quantities following established protocols (Borgonie *et al*. 1995), aliquoted and stored at −20 °C (Schulte *et al*. 2010). In all experiments, *B. thuringiensis* mixtures were used at a final concentration of 1.2 × 10^8^ particles mL^−1^, supplemented with the standard nematode food bacterium, *Escherichia coli* strain OP50, at a final concentration of 2 × 10^9^ cells mL^−1^ (Schulte *et al*. 2010).

### Analysis of male frequencies during experimental host–parasite coevolution

**Figure 1:**
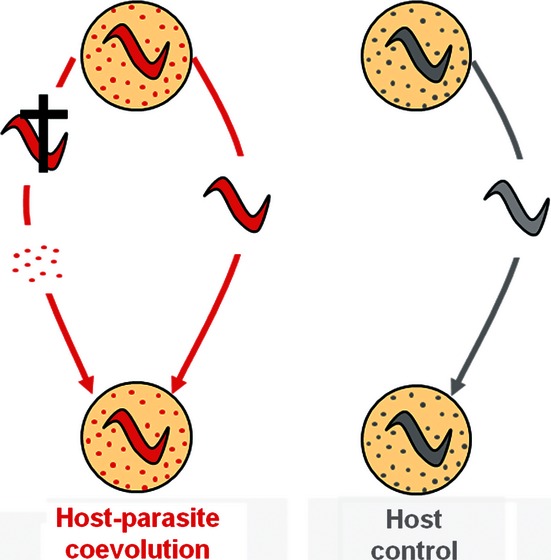
Design of the two evolution experiments. The coevolution treatment is given in red, for which host and parasite were forced to coadapt to each other. The control treatment is indicated in grey and involved evolution of the host under experimental conditions in the absence of the antagonist.

We considered two evolution experiments for our analysis: a previously published experiment (Schulte *et al*. 2010), for which male abundance was recorded but not yet reported, and a new evolution experiment. Both included two evolution treatments for the host (Fig. 1): (1)A coevolution treatment, during which host and parasite were forced to coadapt to each other. This was ensured by transferring only nematodes that survived the interaction (right arrow in left panel, Fig. 1) and only bacteria from dead hosts, which were able to infect and kill hosts (left arrow in left panel, Fig. 1). (2)A control treatment, during which hosts evolved in the absence of pathogens (right panel, Fig. 1). The exact methods for the first evolution experiment were described previously (Schulte *et al*. 2010). The second experiment was based on a modified protocol, resulting in the following differences:

1. Host genetic diversity was higher for the second experiment, which was derived from a larger number of genetically diverse natural isolates (Schulte *et al*. 2010; Teotónio *et al*. 2012).
2. Host population size was larger in the second experiment [500 individuals instead of 120 in the first experiment (Schulte *et al*. 2010)].
3. Even though a small proportion of original nematode genotypes were added in both experiments in regular intervals to minimise random loss of genetic diversity (and thus drift effects), the exact immigration rate differed. In the first experiment, only males were added at 5% of the total population size every fourth host generation, whereas in the second experiment, 2.5% males and 2.5% hermaphrodites were added every second host generation. The average immigration rate for males was nevertheless the same (1.25% per generation).
4. The temperature was higher for the second (19 °C) than for the first experiment (16–18 °C), resulting in generation-time differences (first experiment: 3–4 days; second experiment: 2–3 days).
5. To ensure that a sufficient amount of food was available, host and parasite were transferred once per week in the first, and twice per week in the second experiment.
6. The first evolution experiment comprised 20 replicates per treatment and was performed for 48 host generations, while the second evolution experiment included 10 replicates per treatment and ran over 28 host generations.
7. In the first experiment, male frequencies were recorded at each second transfer step (every fourth host generation), while worms were processed for transfer (Schulte *et al*. 2010). At this point, eggs were isolated from populations and purified using alkaline hypochlorite:NaOH (Stiernagle 2006). Hatched larvae were grown in the absence of pathogens to adulthood and scored for sex. The relationship between males and hermaphrodites thus reflects the primary sex ratio as resulting from male frequency, male mating activity and also the selfing rate of hermaphrodites. In the second experiment, male abundance was scored for frozen samples collected in regular intervals. In particular, a random sample per population was collected at host generations 0, 12, 18, 20, 24 and 28, subjected to alkaline hypochlorite : NaOH treatment, grown under pathogen-free conditions for two generations and then frozen at −80 °C. After completion of the evolution experiment, frozen samples were thawed and grown for one generation without pathogens on peptone-free nematode growth medium (PFM), followed by determination of the sex ratio. Samples were not available for each population at each time point, resulting in 15–20 replicates for the first, and 5–10 replicates for the second evolution experiment per time point and treatment.

All other aspects of the evolution protocol were identical between the two experiments, as described previously (Schulte *et al*. 2010). In both cases, the evolving populations were maintained in ‘wormballs’. These consist of two plastic halves of a ball, filled with a thin PFM layer, which are inoculated with the respective bacteria, followed by addition of worms and closure of the two halves (Schulte *et al*. 2010). Wormballs were used to minimise loss of nematodes, which is typically high on standard petri dishes due to pathogen escape behaviour. The two experiments were performed by different experimenters in different locations (first experiment at Muenster University; second experiment at Tuebingen University, both Germany), resulting in additional random differences. As a consequence, the two evolution experiments are fully independent.

### Analysis of male and hermaphrodite resistance

Survival rate was measured as a proxy for resistance (Schulte *et al*. 2010; Wang *et al*. 2012), using 6-cm PFM plates and 100 μL of pathogenic *B. thuringiensis* (final concentration: 1.2 × 10^8^ particles mL^−1^) and *E. coli* (final concentration: 2 × 10^9^ cells mL^−1^). To assess a possible increase in male mortality due to male–male competition in single sex groups (Gems & Riddle 2000), a control treatment was established using a mixture of non-pathogenic *B. thuringiensis* (final concentration: 1.2 × 10^8^ particles mL^−1^) and *E. coli* (final concentration: 2 × 10^9^ cells mL^−1^). *E. coli* was always added as food to ensure that mortality results from interaction with the pathogen, but not starvation. Prior to experiments, nematode populations were thawed and grown for at least two generations to minimise possible influences of freezing. Ten age-synchronised fourth-stage larvae (L4), either all males or all hermaphrodites, were transferred to assay plates and survival was measured 48 h later for 15 replicates per gender under pathogenic, and five under non-pathogenic conditions. Worms were considered dead if they did not respond to touch.

We also determined host pathogen load (Wang *et al*. 2012), using 6-cm PFM plates with 100 μL of the *B. thuringiensis*–*E. coli* mixture (concentrations as above), followed by addition of either 10 L4 males or 10 L4 hermaphrodites (15 replicates per treatment). Three to five dead worms per plate were transferred after 48 h onto microscopic slides, and photographed for body-size measurements using imagej (http://rsb.info.nih.gov/ij/). Nematodes were repeatedly washed with 15–20 μL H_2_O to remove bacteria adhering to the cuticle. Worms from a given replicate were transferred into 100 μL H_2_O in 1.5-mL tubes. The number of externally associated bacteria that were still present was counted in the surrounding solution using counting chambers (0.1-mm depth). For each replicate, bacteria were then extracted for the group of worms through sonication (six 10-s cycles at 60 Hz), followed by 3-s vortexing with 3- to 4 1-mm zirconia beads. Bacterial numbers were determined using counting chambers as above. The infection load was calculated per size-adjusted nematode and replicate by first subtracting the average bacterial number in the surrounding H_2_O from the average bacterial number extracted from worms per replicate, followed by division by the number and average size of worms in a given replicate. Some replicates were not considered in the final analysis because of insufficient worm numbers (less than three).

### Analysis of male sexual activity and resulting F1 male frequency

We tested whether pathogen exposure influenced male sexual activity, which positively correlates with male abundance due to the androdioecious mating system and competitive superiority of male sperm (LaMunyon & Ward 1995; Lopes *et al*. 2008; Wegewitz *et al*. 2008). For this, 3-cm PFM plates were seeded with 50 μL *B. thuringiensis*–*E. coli* mixture, containing either pathogenic or non-pathogenic *B. thuringiensis* (concentrations as above), followed by addition of three L4 males and three L4 hermaphrodites. After 24 h, the number of scanning and mating males per plate was counted six times *c*. every 45 min. The average of the six counts per plate and male was used as a proxy for male sexual activity (20 and 23 replicates for the pathogen and control treatments respectively). The consequences of male sexual activity on male frequency in the next generation were studied by scoring the sex of 100 randomly chosen F1 offspring (14 and 13 replicates for the pathogen and control treatments respectively).

### Analysis of male and hermaphrodite escape behaviour

*Caenorhabditis elegans* expresses a strong pathogen escape response (Schulenburg & Ewbank 2007). Sex-specific differences in this response might limit availability of males for outcrossing, thus biasing offspring sex ratio. Variation in pathogen escape was studied using a modification of the previously described avoidance assay (Hasshoff *et al*. 2007). Forty microlitres of the *B. thuringiensis*–*E. coli* mixture (concentrations as above) was pipetted into the centre of a 9-cm PFM plate. Test bacteria were mixed with *E. coli* to reduce worm escape caused by absence of food (Hasshoff *et al*. 2007). To minimise return of escaped worms because of food deprivation, an outer ‘food ring’ of 100 μL *E. coli* was pipetted onto the plate's boundaries. Individual L4 nematodes were placed into the middle of the central lawn in the following combinations: one hermaphrodite, one male, 10 hermaphrodites, 10 males or a mixture of five hermaphrodites and five males. These combinations served to assess a possible bias in escape caused by presence of conspecifics, which is known to influence nematode behaviour, especially that of males (Gems & Riddle 2000; Lipton *et al*. 2004; Chasnov *et al*. 2007). Escape was measured towards either pathogenic or non-pathogenic *B. thuringiensis* as the percentage of worms that left the central lawn after 24 h (15 replicates per treatment).

### Analysis of resistance of offspring from self-fertilised or outcrossed hermaphrodites

The resistance of offspring derived from selfed or outcrossed hermaphrodites was studied using two independent experiments, performed by different experimenters in different locations: Experiment 1 at Tuebingen University and experiment 2 at Kiel University. The experiments also differed in the degree of pathogenicity expressed by nematocidal *B. thuringiensis* (see below). Resistance was always compared between F2 offspring of selfed or outcrossed hermaphrodites that went through one generation of selfing during the F1 to minimise potential effects on resistance due to mating, which may, for example, be caused by male-induced harm (Gems & Riddle 1996; Wegewitz *et al*. 2008).

For both experiments, selfed and outcrossed offspring were generated by adding either only hermaphrodites (experiment 1: two hermaphrodites; experiment 2: six hermaphrodites) or a mixture of males and hermaphrodites (experiment 1: four males and two hermaphrodites; experiment 2: three males and three hermaphrodites) to a 3-cm PFM plate inoculated with *E. coli* and non-pathogenic *B. thuringiensis*. Virgin F1 L4 hermaphrodites were transferred to 6-cm PFM plates (with non-pathogenic *B. thuringiensis* and *E. coli*) and allowed to reproduce by selfing. F2 L4 hermaphrodites were either exposed to pathogenic or non-pathogenic *B. thuringiensis*–*E. coli* mixtures on PFM plates (concentrations as above; 10 hermaphrodites per replicate in experiment 1 and six in experiment 2). Freshly thawed pathogenic *B. thuringiensis* were used in experiment 1, whereas in experiment 2 the bacteria were maintained at room temperature for *c*. 2 days, resulting in reduced pathogenicity. Survival was thus scored after 48 h in experiment 1 (25 replicates per treatment) and after 120 h in experiment 2 (40 replicates per treatment).

### Statistical analyses

Variation in male frequency across the evolution experiments was assessed with a general linear model including generation and treatment as fixed factors, and replicate nested within treatment as random factor. The Wilcoxon rank sum test was used to assess variations between males and hermaphrodites or between pathogen and control treatments in host resistance, male sexual activity and escape behaviour. In case of multiple testing for escape behaviour analysis, we adjusted significance levels using the false discovery rate (Benjamini & Hochberg 1995). The difference in either the mean or variance of survival between outcrossed vs. selfed F2 offspring was examined with either the Wilcoxon rank sum or the Brown–Forsythe test, which both take into account that the data was nonparametric. Statistical analyses were performed using jmp in 9.0 (SAS Institute Inc.), and graphs were generated using SigmaPlot 12.0 (Systat Software Inc.).

## Results

**Figure 2:**
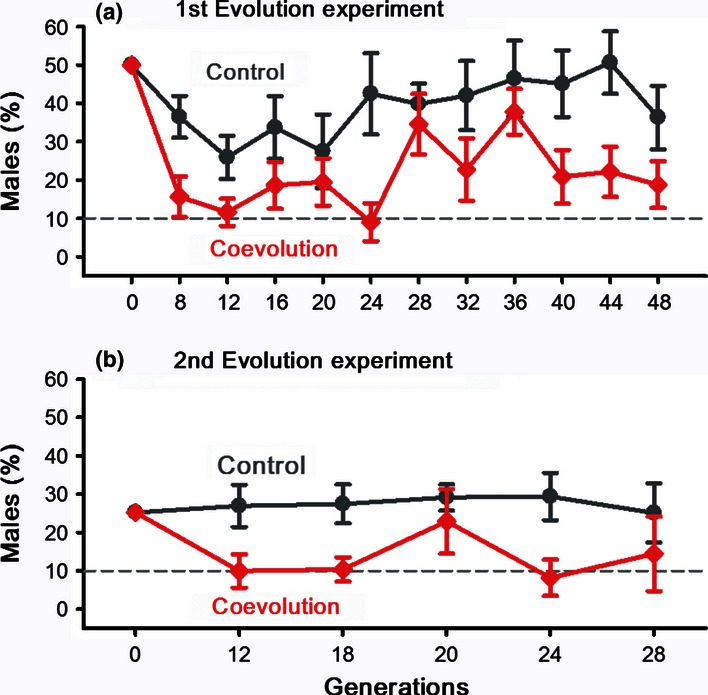
Male frequency over time across two fully independent evolution experiments (a, b). The coevolution treatment is shown in red and the control in grey (Fig. 1). The results are given as means with 2 × standard errors of the mean (SEM), including per time point and treatment 15–20 replicates for the first and 5–10 replicates for the second evolution experiment. The dashed grey line indicates a male frequency of 10%.

Male *C. elegans* frequencies were scored during two fully independent evolution experiments, which both comprised a coevolution and a control treatment (Fig. 1). Both experiments consistently demonstrated that coevolution leads to significantly reduced yet comparatively stable male frequencies of 10–20% if compared with control conditions (*F* > 10, *P* ≤ 0.0021; Fig. 2; Fig. S1 and Table S1).

**Figure 3:**
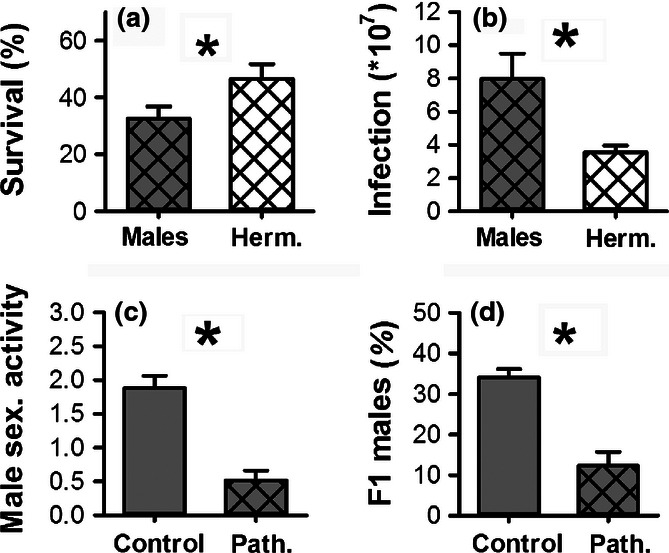
Male pathogen resistance and sexual activity. (a) Host survival on pathogenic *B. thuringiensis*. (b) Host infection load. (c) Male sexual activity, measured as the average number of males either courting or copulating with a hermaphrodite. (d) Resulting male frequency in the F1 generation. Bars show means with 2 × SEM; asterisks indicate significant differences.

To identify possible causes for the lower yet stable male frequencies, separate focused experiments were performed, using nematodes from the genetically diverse starting population of the second evolution experiment. We first tested whether lower male frequencies might be due to lower male pathogen resistance. This hypothesis was assessed by scoring survival and infection load as proxies for pathogen resistance. Indeed, male survival was lower than hermaphrodite survival in the presence of pathogenic *B. thuringiensis* (*Z* = 2.06, *P* = 0.039; Fig. 3a), but not under non-pathogenic conditions (*Z* = 0, *P* > 0.99; average male and hermaphrodite survival was 0.967 ± 0.033 and 0.956 ± 0.044 respectively). Similarly, males were more heavily infected than hermaphrodites (*Z* = 2.22, *P* = 0.026; Fig. 3b).

We next asked whether pathogen exposure affects other traits known to influence male frequencies. One of these traits is male sexual activity, which affects male abundance because of the androdioecious mating system. Indeed, the average number of males that either mated or courted hermaphrodites was lower under pathogenic than control conditions (*Z* = 4.43, *P* < 0.001; Fig. 3c). Moreover, the decreased male sexual activity translated into lower male numbers in the offspring generation (*Z* = 3.81, *P* < 0.001; Fig. 3d).

**Figure 4:**
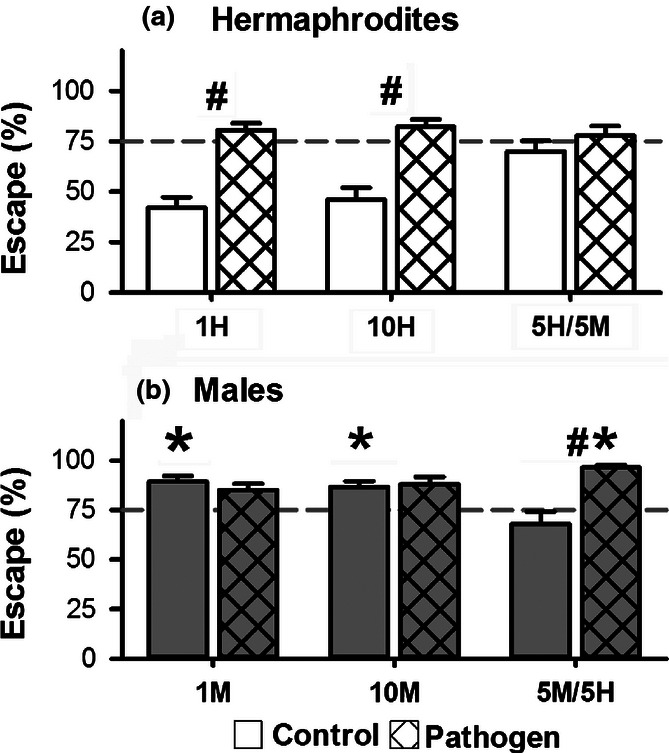
Escape behaviour for (a) hermaphrodites and (b) males. Escape was evaluated in response to either pathogens (patterned boxes) or non-pathogens (solid boxes) for single worms, 10 worms of the same gender or 10 worms of 50% hermaphrodites (H) and 50% males (M). Bars show means with 2 × SEM. Asterisks in (b) indicate the treatments for which males and hermaphrodites show significant differences. Crosses in (a) and (b) point to significant differences between pathogen and control treatments for a particular sex and group composition. The dashed grey line indicates an escape of 75% for comparisons across panels.

Another relevant trait is male-specific behavioural escape from pathogens, which might limit availability of males for outcrossing. Our analysis of this trait revealed an intricate behavioural response contingent on gender type, presence of the pathogen and potential mates (Fig. 4). In the treatments with only one of the genders, male nematodes left non-pathogenic bacteria more frequently than hermaphrodites (*Z* > 4.4, *P* < 0.0001, Fig. 4), whereas both avoided pathogens at similar rates (*Z* < 0.9, *P* > 0.3, Fig. 4). This observation is consistent with previous reports of high male roaming activity in the absence of hermaphrodites, aimed at finding mates (Lipton *et al*. 2004). Importantly, in mixed-gender groups that reflected the conditions of the evolution experiment, almost 100% of males escaped from pathogens, significantly more than observed for hermaphrodites (*Z* = 3.57, *P* = 0.0003; Fig. 4).

**Figure 5:**
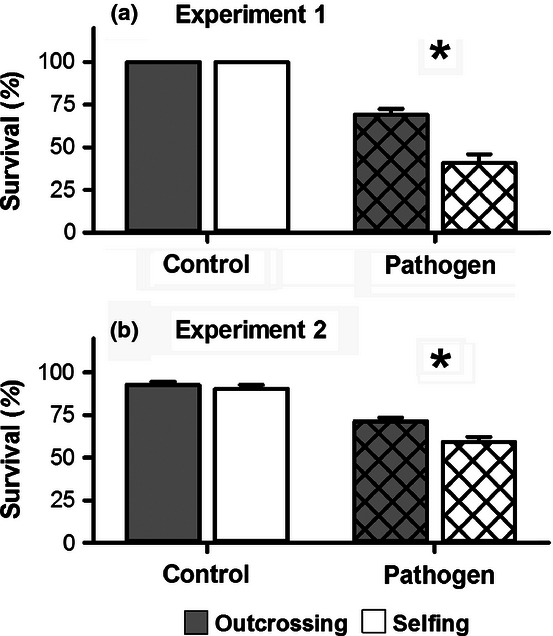
Survival of progeny from outcrossed vs. selfed grandparents for two fully independent experiments (a, b). In both experiments, survival was measured under either pathogenic or control conditions for the F2 generation, derived from either outcrossed or selfed grandparents, including one generation of selfing in the F1 to remove potential parental effects caused by mating. Bars represent means with 2 × SEM and asterisks indicate significant differences in the mean survival rate.

We finally asked whether males – in spite of the above selective disadvantages – may still provide a selective benefit by increasing offspring resistance after male-dependent outcrossing. Offspring were assayed in the F2 generation after having gone through one generation of selfing to remove potential maternal effects associated with outcrossing. The comparison was performed twice by different experimenters in different locations using small differences in the exact protocol. Both comparisons consistently demonstrated that outcrossing led to a significant increase in the mean but not the variance of F2 offspring resistance (Comparisons of the means, *Z* > 2.2, *P* < 0.025; Comparisons of the variance, *F* < 3.1, *P* > 0.08; Fig. 5). No significant differences were observed for the controls (in all cases, *P* > 0.17; Fig. 5).

## Discussion

Our study provides experimental evidence for the potential importance of sex differences in host defence during host–parasite coevolution. It is based on the observation of male frequencies in two fully independent evolution experiments and a subsequent systematic analysis of the selective advantages and disadvantages of males during their interaction with parasites. The experiments were performed with genetically diverse starting populations and, thus, consider natural variation in *C. elegans*, contrary to most work in this species. We consistently found a reduction in male abundance when *C. elegans* was experimentally forced to coadapt with its microparasite *B. thuringiensis*. As male numbers were scored under pathogen-free conditions for offspring of parents from the evolution experiments (first-generation offspring for the first evolution experiment; third-generation offspring for the second evolution experiment) and as *C. elegans* sex ratio is considered to directly reflect outcrossing rates (e.g. Teotónio *et al*. 2006, 2012; Lopes *et al*. 2008; Morran *et al*. 2011), we conclude that coevolution causes a decrease in outcrossing frequency. This finding is in apparent contrast to the Red Queen hypothesis, which predicts increased outcrossing rates during coevolution. Indeed, this prediction was recently confirmed in a related *C. elegans* coevolution experiment (Morran *et al*. 2011). This experiment had a distinct focus and differed in several aspects from our approach such as usage of: (1)A different pathogen, *S. marcescens*; (2) *C. elegans* populations with a different genetic background that were additionally genetically manipulated to express alternative reproductive modes; and (3)A different selection environment, which forced worms to either resist or escape *S. marcescens* in the pathogen treatments (Morran *et al*. 2011). The study identified an increase in male frequency during coevolution, suggesting increased outcrossing. Outcrossing during coevolution provided a selective advantage because it associated with increased resistance (Morran *et al*. 2011, 2012).

In our case, reduced male frequencies are explained by their lowered ability to cope with pathogenic *B. thuringiensis*. We identified three key characteristics that are likely of relevance in this context: reduced male resistance against *B. thuringiensis*, decreased male sexual activity in the presence of the pathogen and possibly enhanced male escape behaviour. The decreased sexual activity might have been a direct consequence of lower male resistance, because it may constrain male performance during the mating process, starting with initial perception of hermaphrodite sex pheromones, followed by male movement towards hermaphrodites, localisation of the vulva, male spicule insertion into the vulva and subsequent sperm transfer (Barr & Garcia 2006). Nevertheless, low sexual activity should have still amplified the effect on male abundance through the reduced production of males in the offspring generation (e.g. LaMunyon & Ward 1995). Furthermore, the enhanced escape behaviour of males may have additionally biased sex ratio if it reduced male–hermaphrodite encounter and thus mating rates. Note that experimental evolution was performed in wormballs (Schulte *et al*. 2010) that minimise, but may not fully exclude nematode escape responses against pathogens.

Intriguingly, previous experimental analysis of *C. elegans* sex-ratio evolution demonstrated that it is the reduction in male mating ability and the subsequent lower male abundance, which lead to rapid loss of males from nematode populations (comparison of strains N2, JU440 vs. CB4856, PX174, and strain N2 vs. CB4856 respectively in Teotónio *et al*. 2006; Wegewitz *et al*. 2008). The importance of male mating ability on male abundance is corroborated by mathematical models (e.g. Cutter *et al*. 2003). These findings strongly suggest that the male disadvantages recorded here and especially their combination should have similarly caused male frequencies to drop to values close to zero in the coevolving host populations. This is still true if a low male immigration rate of 1.25% per generation is taken into account, which was part of the design of both evolution experiments to prevent random loss of genetic variation (see Materials and Methods): Previous evolution experiments with *C. elegans* populations of similar census size (100 individuals) demonstrated that a comparable male immigration rate (1%) led to an invasion success of below 3%, even if immigrant males had high mating ability (males of genotype CB4856, Wegewitz *et al*. 2009). Consequently, based on the currently available information, the most parsimonious explanation for the observed lowered yet stable maintenance of males at a frequency of more than 10% is that males also provide a selective benefit in the presence of parasites.

One possible male benefit can be derived from the Red Queen hypothesis, if male-dependent outcrossing increases offspring resistance. The Red Queen hypothesis would thus still apply irrespective of the lower male abundance during coevolution. In this case, male benefits may be due to a so-called short-term effect (e.g. in case of heterozygote advantage and the increased abundance of heterozygotes through outcrossing) and/or a long-term effect (e.g. the increase in recombination leads to a break-up of alleles with negative association, Agrawal 2006). The former would result in a higher mean and the latter in a higher variance of pathogen resistance (Agrawal 2006). Our results clearly support the first alternative (Fig. 5). Therefore, we identified an indirect benefit for males. This benefit seems to stem from a short-term advantage of outcrossing, for example, as a consequence of heterosis in the presence of parasites. Our finding of a selective advantage of outcrossing is thus in general agreement with the *C. elegans* coevolution experiment by Morran and co-workers (Morran *et al*. 2011, 2012) – in spite of the contrary observations on male frequencies.

In conclusion, our systematic analysis demonstrated that male hosts can provide both a direct selective disadvantage and also an indirect selective benefit in the presence of parasites. The direct disadvantage is most likely a consequence of intraspecific selective constraints (e.g. sexual selection), whereas the indirect benefit is important during interspecific selection among hosts and parasites. Our results thus point to a conflict among intra- and interspecific selective constraints. These opposing selective constraints are likely to have acted jointly during our coevolution experiments and should thus be responsible for the observed reduced yet stable male frequencies.

What are the possible consequences of these two opposing selective constraints on host–parasite coevolution? Two main alternatives are conceivable. The first possibility is that reduction in male performance under pathogen pressure may result from a general mechanism. For example, all males – irrespective of the underlying genotype – may reduce mating activity when parasites are present. Chance then determines which male genotypes achieve successful matings. In the long term, low male mating activity and low outcrossing rates should decrease genetic diversity and thus the adaptive potential of the host. This effect may, however, be compensated for, if outcrossed rather than selfed offspring shows higher resistance, as shown in this study. In this case, reduced outcrossing rates may still allow the host to adapt to the evolving antagonist, in general agreement with previous theoretical models (Agrawal & Lively 2001), although adaptation may not be as fast as under high outcrossing frequencies. The second possibility is that reduction in male performance may be genotype specific. For example, male host genotypes vary in resistance towards parasites. In this case, only the resistant genotypes mate, thus resistance alleles should spread faster through the host population, leading to high adaptation rates. This effect may even be enhanced if outcrossing on its own produces an increase in offspring resistance. In case of *C. elegans*–*B. thuringiensis* coevolution, our published results of the first evolution experiment (Schulte *et al*. 2010) showed that coevolved hosts increased in pathogen resistance, although the increase appeared moderate. Moreover, male frequencies remained low and did not increase again across time (Fig. 2), which they should if outcrossing leads to higher resistance in both genders, as specifically expected under the second alternative above. Therefore, this particular case seems to be more consistent with the first alternative.

How widespread is the interference of these selective forces during host–parasite coevolution? Irrespective of which gender shows lower immunocompetence, the difference between the sexes leads to a bias in the availability of genotypes in one sex. This influences the potential for adaptation in response to outcrossing (see discussion above). For *C. elegans,* it seems to depend on the pathogen, which gender is more or less immunocompetent. For example, in contrast to our results, male *C. elegans* showed higher resistance than hermaphrodites towards a fungal pathogen (van den Berg *et al*. 2006). The coevolution experiment by Morran and co-workers revealed increased male frequencies during coevolution and higher pathogen resistance for outcrossed populations. This suggests high male resistance, although this trait was not analysed for each sex separately, but rather for entire populations with variable sex ratios (Morran *et al*. 2011, 2012). More generally, sex-specific variation in immune defence is expected to be common among animals. A reduction in male immunocompetence is predicted by Bateman's principle (Rolff 2002), and it is indeed observed across a large variety of vertebrate and invertebrate taxa (reviewed in Nunn *et al*. 2009; see also Aisenberg & Peretti 2011; Roth *et al*. 2011). Sex differences in immunity may also result from other intraspecific influences such as higher parasite exposure of one sex due to differential dispersal behaviour or host sex-dependent variation in parasite virulence (Stoehr & Kokko 2006; Restif & Amos 2010). For the full appreciation of host–parasite coevolutionary dynamics, it is therefore essential to take into account these widespread sex-specific variations in immunocompetence.
