# Supplementary material for: Sex differences in host defence interfere with parasite-mediated selection for outcrossing during host–parasite coevolution
Source: Ecol Lett. 2013 Jan 10;16(4):461–8. doi: 10.1111/ele.12068 (PMC3655609; doi:10.1111/ele.12068)
Supplement: Supplementary file 1 [file ele0016-0461-SD1.doc]

**Supplementary Figure S1. Male frequencies over time for individual replicate populations.**

**
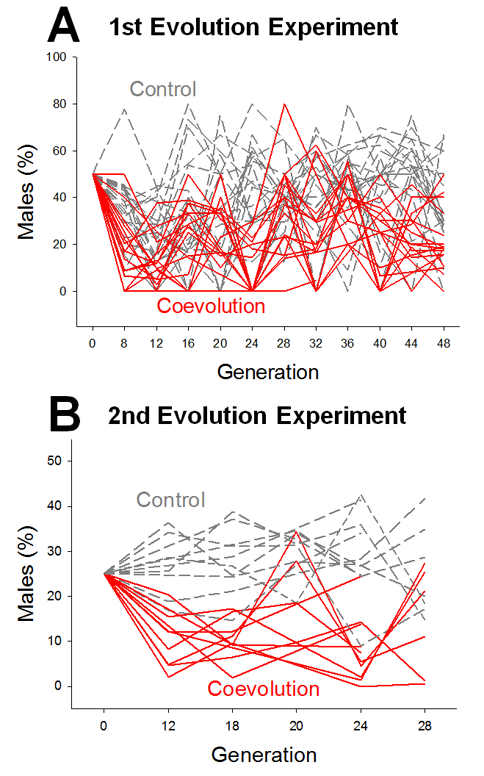
**

**Supplementary Figure 1**. Percentage of males across time for the individual replicate populations of the two evolution experiments (**A, B**). In both cases, solid red lines indicate host-parasite coevolution and gray dashed lines control evolution.
