# Supplementary material for: Sex differences in host defence interfere with parasite-mediated selection for outcrossing during host–parasite coevolution
Source: Ecol Lett. 2013 Jan 10;16(4):461–8. doi: 10.1111/ele.12068 (PMC3655609; doi:10.1111/ele.12068)
Supplement: Supplementary file 2 [file ele0016-0461-SD2.doc]

**Supplementary Table S1. Results of the statistical analysis of male frequencies for the two evolution experiments***

| **Experiment** | **Factor** | ***df*** | ***F*** | ***P*** |
| --- | --- | --- | --- | --- |
| 1 | Treatment | 1 | 16.11 | **< 0.0001** |
|  | Generation | 10 | 7.57 | **< 0.0001** |
|  | Treatment x Generation | 10 | 2.07 | **0.0026** |
| 2 | Treatment | 1 | 10.25 | **0.0021** |
|  | Generation | 4 | 1.87 | 0.1298 |
|  | Treatment x Generation | 4 | 1.31 | 0.2771 |

* For each of the two evolution experiments (as indicated by first column), the analysis was based on a mixed general linear model, using log transformed data and the restricted maximum likelihood (REML) approach, as implemented in the program JMP IN 9.0 (SAS Inst. Inc.). The model consisted of treatment and generation as fixed factors and replicate population nested within treatment as random factor. The latter factor acknowledges that the measurements for one particular replicate population are related across time. Host generation 0 was identical between treatments and thus not included in the analysis. The table shows the results for fixed factor effect tests using F statistics (see details in the manual to JMP IN 9.0, SAS Inst. Inc.). Abbreviations: *df*, degrees of freedom; *F*, F ratio test statistic, and *P*, probability. Significant probabilities are given in bold. The factor treatment has the strongest significant effect on male frequencies in both evolution experiments.
